# Practical guidance for running late-phase platform protocols for clinical trials: lessons from experienced UK clinical trials units

**DOI:** 10.1186/s13063-022-06680-4

**Published:** 2022-09-06

**Authors:** Sharon B. Love, Fay Cafferty, Claire Snowdon, Karen Carty, Joshua Savage, Philip Pallmann, Lucy McParland, Louise Brown, Lindsey Masters, Francesca Schiavone, Dominic Hague, Stephen Townsend, Claire Amos, Annabelle South, Kate Sturgeon, Ruth Langley, Timothy Maughan, Nicholas James, Emma Hall, Sarah Kernaghan, Judith Bliss, Nick Turner, Andrew Tutt, Christina Yap, Charlotte Firth, Anthony Kong, Hisham Mehanna, Colin Watts, Robert Hills, Ian Thomas, Mhairi Copland, Sue Bell, David Sebag-Montefiore, Robert Jones, Mahesh K. B. Parmar, Matthew R. Sydes

**Affiliations:** 1 MRC Clinical Trials Unit at UCL, 90 High Holborn, London, WC1V 6LJ UK; 2 The Institute of Cancer Research, London, SW7 3RP UK; 3 Cancer Research UK Clinical Trials Unit, Level 0 The Beatson West of Scotland Cancer Centre, 1053 Great Western Road, Glasgow, G12 0YN UK; 4 Cancer Research UK Clinical Trials Unit (CRCTU), Institute of Cancer and Genomic Sciences, University of Birmingham, Edgbaston, Birmingham, B15 2TT UK; 5 Centre for Trials Research, Cardiff University, Neuadd Meirionnydd, Heath Park, Cardiff, CF14 4YS UK; 6 PHASTAR, Bollo Lane, London, W4 5LE UK; 7 University of Oxford, Oxford, OX3 7DQ UK; 8 The Institute of Cancer Research, London, SW3 6JB UK; 9 Cancer Research UK Clinical Trials Unit, Institute of Cancer and Genomic Sciences, College of Medical and Dental Sciences, University of Birmingham, Birmingham, B15 2TT UK; 10 Comprehensive Cancer Centre, King’s College London, Guy’s Campus, New Hunt’s House, Room 2.36b, London, SE1 1UL UK; 11 Institute for Head and Neck Studies and Education, University of Birmingham, Birmingham, B15 2TT UK; 12 Institute of Cancer and Genomic Sciences, University of Birmingham, Birmingham, B15 2TT UK; 13 Doll Building, CTSU, Nuffield Department of Population Health, University of Oxford, Old Road Campus, Roosevelt Drive, Oxford, OX3 7LF UK; 14 Centre for Trials Research, Cardiff University, Neuadd Meirionnydd, Heath Park Way, Cardiff, CF14 4YS UK; 15 Paul O’Gorman Research Centre, Gartnavel General Hospital, Glasgow, G12 0YN UK; 16 Clinical Trials Research Unit (CTRU), Leeds Institute of Clinical Trials Research, University of Leeds, Leeds, LS2 9JT UK; 17 Level 4 Bexley Wing St James Institute of Oncology, Leeds, LS9 7TF UK; 18 Beatson West of Scotland Cancer Centre, Glasgow, UK

**Keywords:** Platform protocols, Trial conduct, Multi-arm multi-stage trials, Umbrella trials, Basket trials, Stratified medicine, Complex innovative designs, Methodology

## Abstract

**Background:**

Late-phase platform protocols (including basket, umbrella, multi-arm multi-stage (MAMS), and master protocols) are generally agreed to be more efficient than traditional two-arm clinical trial designs but are not extensively used. We have gathered the experience of running a number of successful platform protocols together to present some operational recommendations.

**Methods:**

Representatives of six UK clinical trials units with experience in running late-phase platform protocols attended a 1-day meeting structured to discuss various practical aspects of running these trials. We report and give guidance on operational aspects which are either harder to implement compared to a traditional late-phase trial or are specific to platform protocols.

**Results:**

We present a list of practical recommendations for trialists intending to design and conduct late-phase platform protocols. Our recommendations cover the entire life cycle of a platform trial: from protocol development, obtaining funding, and trial set-up, to a wide range of operational and regulatory aspects such as staffing, oversight, data handling, and data management, to the reporting of results, with a particular focus on communication with trial participants and stakeholders as well as public and patient involvement.

**Discussion:**

Platform protocols enable many questions to be answered efficiently to the benefit of patients. Our practical lessons from running platform trials will support trial teams in learning how to run these trials more effectively and efficiently.

**Supplementary Information:**

The online version contains supplementary material available at 10.1186/s13063-022-06680-4.

## Background

There is increasing awareness of the potential efficiencies of more comprehensive designs for clinical trials protocols which test multiple primary clinical questions within a single protocol [1–4]. These designs, which include, multi-arm multi-stage (MAMS), platform, basket, umbrella, and master protocols, can ask multiple questions in one protocol with a reduction in time and/or cost compared to separate two-arm protocols. Here, we use “platform protocols” to cover this wide range of designs. Since the benefits from efficient designs will be greater in absolute terms in late-phase designs, we focus towards randomised phase IIb and III trials addressing more than one primary question and which are designed to include an adaptive element.

The potential benefits of the platform protocol approach for participants may be considerable including, allowing participants to contribute to more than one question or providing participants with more access to novel treatments, yet relatively few clinical trials units have designed, conducted, and analysed platform protocols [5]. Platform protocols require increased knowledge and clearer guidance for the trials community to ensure effective implementation; practical experience papers can help in this regard [6–12]. To reflect on important practical lessons across trials, we convened a workshop for representatives associated with six UK Clinical Research Collaboration (UKCRC)-registered trials units who had led late-phase platform protocols. Here, we draw together lessons based on these practical experiences.

Three different examples of platform protocols are highlighted in Table 1, and further details and examples are given in supplementary file 1. Our recommendations on operationalisation are presented broadly in the order of a trial’s life cycle.

**Table 1:** Three examples of late-phase platform protocols

**PLATO** (ISRCTN88455282) is a platform protocol, labelled as an “umbrella” trial, in anal cancer, a rare tumour with 1000 UK diagnoses per year [13]. Platform approaches help to address recruitment challenges posed by rare diseases, allowing trials to be performed that would not be viable on their own. PLATO comprises three individually powered sub-trials: the three-arm ACT5 for high-risk disease, the most common presentation; the smaller two-arm ACT4 for intermediate risk disease; and the non-randomised ACT3 for low-risk disease which would likely never have run on its own. Combination in a single platform allowed evaluation of a broader set of clinical and translational objectives (see Additional file 1 for flow diagram)
**Add Aspirin** (ISRCTN74358648) is a basket trial assessing the use-and-duration of aspirin in four tumour-specific randomisations. Each is individually powered with a primary outcome measure relevant to the disease setting. The overarching platform allowed a cohesive and consistent approach to complementary studies, with the potential to increase impact of the results. Overall survival will be assessed across the tumour-specific cohorts and increased power for secondary research questions (such as assessing the effect of different aspirin doses) will also be achieved by combining the cohorts. Release of primary results (which will be available at different times for different cohorts) will need to be carefully managed, considering the impact on ongoing cohorts. Efficiencies are derived from having a single protocol requiring only a single approval at both the global and local (site) level; however, protocol amendments can take longer to implement due to the need to consult multiple groups/stakeholders and ensure the impact of any changes are considered for each tumour-specific cohort. Whilst there is only one DSMC and one TMG, the latter functions mostly as a number of separate subgroups (each the size of a more traditional TMG) (see Additional file 1 for flow diagram)
**CompARE** (NCT04116047) is a phase III multi-arm multi-stage (MAMS) trial assessing treatments for patients with high-risk oropharyngeal cancer. The trial started with four arms, three research, and one control. During the trial, one further research treatment was added before assessment and recruitment to three of the research arms has been completed (see Additional file 1 for flow diagram)

## Methods

Representatives of trials run from six UKCRC-registered clinical trials units with experience in running late-phase clinical trials testing multiple primary research hypotheses were invited to attend a 1-day meeting in London in March 2019. The expertise of the 26 attendees included operational, clinical, statistical, and methodological perspectives. Although no patient and public involvement (PPI) representatives attended the meeting, we have incorporated input from 2 PPI co-authors subsequently. The meeting and discussions were structured to focus on areas of a trial’s life cycle and attendees focused on selected processes and important issues that might be either harder to implement in, or specific to, platform protocols compared to a traditional parallel two-arm late-phase trial. The emphasis was on operational rather than statistical, clinical, or ethical issues, which are covered elsewhere [1, 2, 14]. Trial-specific plenary presentations drove full-group and break-out discussions to identify issues of which other researchers running such trials should be aware. The key points from the discussion have subsequently been iterated and shaped through into the guidance summarised in Table 2 and described in further detail in the “Results” section.

**Table 2:** Summary of recommendations

Area in results section	Recommendation
1. Communication	Trial descriptions should be tailored to each audience and each occasion: this is to reduce the (perceived) complexity of a platform protocol where a patient may contribute to only one part of the trial
	Clear diagrams are essential to express: (a) Each individual comparison (b) The currently-recruiting comparisons (c) The changes to the open comparisons over time
	All patient-facing and ethics committee text should clearly describe: (a) The current trial (b) The information which is relevant for the specific participant group
	Avoid overloading participants and recruitment teams by using a staged consent process
	A trial with a big central team should provide one person as a dedicated point of contact to each site
2. Funding	Funding is likely (to need) to come from multiple sources
	Each contribution to funding should aim to include a contribution to the overall infrastructure and the common delivery of the platform
	Express to funders both the savings (time, patients, cost) and/or gains (additional scientifically important questions) of using a platform protocol over separate trials
3. Protocol	Choose the most future-proofed option between modular or single approach to protocol development
	When choosing whether patients not meeting the eligibility criteria for one comparison could be randomised to other comparisons, consider: (a) Implications on recruitment (b) Generalisability of findings (c) Practical implementation at sites
	Explicitly state in the protocol from the outset that future comparisons will be incorporated into the protocol if appropriate
	Early engagement and ongoing communication with the regulator is essential
	Aim for review by an ethics committee with previous experience and training in platform protocols
4. Database and randomisation system	The database needs to be flexible and scalable
	Modular database design with shared elements is preferable if the current or future arms may differ in terms of the information required
	Allow for sufficient data management time in each grant. Platform protocols are more efficient in real-time results and input may be required over a shorter time than for any one trial
	Ensure choice of randomisation system can incorporate any necessary future amendment (e.g. to eligibility, weightings and stratification factors)
5. Patient and public involvement	Early PPI input improves the design
	Support PPI to understand design implications, particularly in adding new comparisons
	PPI participation helps the trial team with explanations to ethics committees
	Comparison-specific PPI representation can give a more manageable workload for PPI members and enable trial teams to better support PPI members
6. Contracts	Contracts must allow for the longevity of platform trials
	Platform protocols are likely to have more external collaborators so allow time for set-up and agreement
7. External trial oversight	Trial oversight committees must expect greater longevity and a considerable workload over time and per meeting, particularly if a platform protocol has many comparisons
	Members must be experienced, and any handover should aim to include an overlap period
8. Trial Management Group	The responsibilities of large TMGs may usefully be delegated to specific sub-committees, each responsible for components of the platform
	A dedicated lead for each comparison could better support the chief investigator and trial team in development, conduct, and reporting, e.g. comparison CI and comparison co-CI
9. Trial staffing	Flexible staffing allows for more staff at time of higher need
	Allow more senior time to manage a bigger team
10. Data management	Data cleaning and checking must be an ongoing process rather than analysis driven, so that all arms are updated fairly
	Create a dedicated site advisory team including site representatives
11. Statistical considerations	Choice of single or separate SAPs is driven by which comparisons will be analysed and reported and when they are to be reported (contemporaneously or at different times)
	Need to have a senior statistician unblinded and involved in analyses during the trial and another senior statistician who is blinded and unaware of the accumulating data analysis
12. Safety	SAEs must be assessed against the expected events for each of the treatments. Multiple research treatments require additional time at sites and during safety review
	Careful management of reference safety information is required with multiple treatments
	Multiple groups often need to be notified of SAEs
13. Training	Regularly update training materials and documentation
	Make training materials simple and inspiring to avoid site fatigue in long-term protocols with multiple new comparisons and amendments
	Make clear to staff that recruitment need not be paused around intermediate analysis
14. Reporting	Aim to give results from across the protocol to all patients, with a contextualising preface specific to their allocation/comparison
	Ideally ask whether participants want findings from only “their” comparison, from all comparisons, or no results
	CONSORT extensions for multi-arm randomised controlled trials and adaptive trials provide pertinent guidance
	Write a publication plan to minimise scheduling clashes for limited staff time
	Discuss authorship principles at comparison set-up. Authorship need not be the same for each primary comparison but all relevant names, including all relevant funders, must be noted
15. Adding and closing comparisons	Adding comparisons requires agreement from oversight committees, regulators, and assent from sites
	Consider using a checklist in deciding whether to add to the current trial or start a new trial [7]
	Aim to close down elements of each comparison as soon as practicable rather than leaving all comparisons open
16. Maintaining relevant control arm treatment	Be prepared for the standard-of-care to change over the lifetime of a platform trial
17. Onward data sharing and re-use	Consider whether data from reported comparisons can be shared on appropriate data release request without compromising ongoing comparisons or planned analyses

**Fig. 1:**
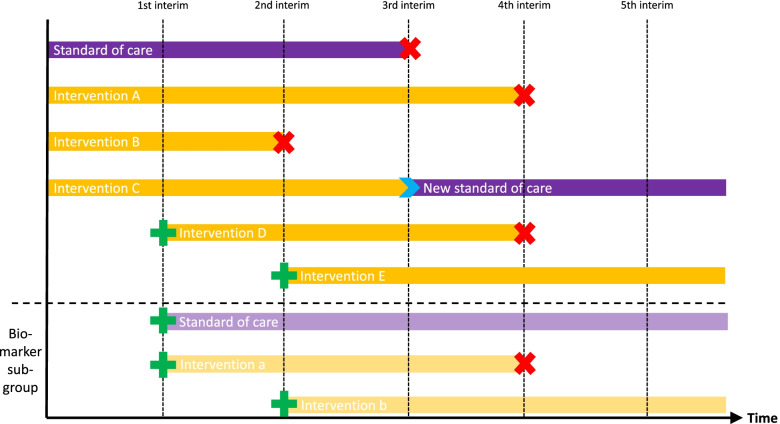
A platform protocol. Green plus signs show where a new arm is started during the trial, and red cross signs show when recruitment to an arm is stopped

Where appropriate, some examples have been anonymised to maintain confidentiality. Although the expertise was not limited to oncology trials, the examples used in the text are exclusively oncology trials as these were the longer running trials within the attendees. We have referred to “a new comparison” to capture the amendment of a protocol to incorporate a new randomised comparison, whether adding a new intervention arm and extending recruitment to an existing control arm or the addition of both a new research arm and a new control intervention (see Fig. 1).

## Results—findings and recommendations

The concepts and guidance are presented approximately in the order of the life cycle of a traditional trial and the key points are summarised in Table 2.

### Communication

When explaining a potentially complex trial design, it is essential to consider the intended audiences (e.g. potential participants, clinicians, regulators, ethics committees, the public) and tailor the information appropriately: what does this audience already know about trial designs, what are they most likely interested in or need to know, and what sort of language is appropriate?

A clear diagram of the trial design and the flow of what could happen to each participant in the trial should help all audiences, although the level of detail required will vary. Labels such as “platform,” “basket,” or “umbrella” lack universally accepted definitions and some example protocols described trials that fall across multiple categories. We recommend greater emphasis on developing clear graphical depictions of the design rather than on relying on terminology (see Supplementary file 1 for examples).

All text for potential participants should explain the relevant components of the trial. Patient representatives on STAMPEDE (ISRCTN78818544) often remind the Trial Management Group (TMG) that, whilst design features may fascinate many researchers, individual patients are more likely interested in the aspects that directly affect them. This can be addressed by making clear the treatment groups to which they could potentially be randomised and de-emphasising discussion of treatments assessed in other elements of the protocol. Similarly, ethics committees and funders are primarily interested in what the design can achieve, rather than its novelty [15, 16].

A particular format that was found to be useful was a staged informed consent process. This can avoid overloading potential participants with unnecessary information and ease the burden on those discussing consent. Potential participants need to be given sufficient, applicable detail to enable an informed decision at that stage. For example, at the first stage of consent participants could be given detail about being tested for a biomarker as screening for entry and at the second stage of consent, when the biomarker is known, participants could be given detail about which treatments they may be randomised between.

Communication between sites and the trial teams is key and trial teams for such protocols can be large. Therefore, we recommend dividing sites across operations members of trial teams so site teams have a dedicated person to contact centrally with questions.

### Funding

Researchers need to recognise that funding may need to be sought from multiple sources across the life of a platform protocol and this multiple collaboration will take more time than if there was one funder. Cancer examples (e.g. CompARE, FLAIR, STAMPEDE, plasmaMATCH, AML LI-1) started with both a research trial infrastructure grant from a cancer charity (Cancer Research UK or Blood Cancer UK) and support in some form from an industry partner, e.g. FLAIR with Jansen; STAMPEDE with Pfizer, Novartis and Sanofi; plasmaMATCH with AstraZeneca and Puma; AML LI-1 Cyclacel, Ambit and Sunesis), together with flexible use of core MRC funding for STAMPEDE. Each new comparisons must come with sufficient funding and include a contribution to fund the overall infrastructure. For example, new comparisons in these trials have included collaboration with and support from Abbvie, Astellas, AstraZeneca, Bayer BCTI, Synta, Cell Therapeutics, Karyopharm, and Janssen, demonstrating that key international pharmaceutical companies are open to supporting comparisons within ongoing platform protocols.

Protocols determined upfront to be platform protocols are still relatively new for most charitable funders. It is important in each application to make clear the potential savings in using this efficient design in terms of time, patient numbers and cost versus running separate trials, and the potential gain in terms of answering additional questions, even if the form does not explicitly ask for this information. For example, STAMPEDE, a MAMS platform protocol in prostate cancer, will report over a 20-year period comparisons of 10 potential treatments against the contemporary standard-of-care, each powered to detect a benefit in overall survival. To achieve this through 10 traditional 2-arm trials delivered sequentially might be expected to take more than 3 decades and involve many more patients receiving control therapies.

Drawing new comparisons into an existing platform feels operationally more efficient than opening a new traditional trial which competes for patients or recruits subsequently, but this choice requires wide support from the research community within the disease area. Some of the efficiency is derived from the same team building out of the same protocol and statistical analysis plans. However, the CompARE, FLAIR, and STAMPEDE teams estimated time from initial meeting to discuss an additional arm until first site activated for the new comparison was a median of 30 months (range 12–35 months). These teams followed the usual due diligence processes in assessing the importance of the clinical question and to consider potential issues in operationalisation. Timelines can be impacted by infrequent meetings of funding review panels. Therefore, getting to first site activated may be no quicker than for a separate standalone traditional two-arm randomised controlled trial (RCT), but getting to full speed of recruitment is often quicker. For example within STAMPEDE, the first 80 sites took 5 years to open, whereas for the first added comparison, it took less than 3 months to get to first patient entered in 80 sites.

Due to the occasional need for many tasks to be done at the same time (for example, analysing and closing out one arm whilst opening a new comparison) or the longer time required to design such trials, there is often a need at some point in a platform protocol for funding outside of a grant (for example through a trials unit infrastructure award). Trials units should be aware of this risk before taking on a platform trial and funders should consider accepting funding this as part of their costing model.

### Protocol

Careful protocol development is essential for effective conduct of a platform protocol. Two structures have arisen: modular and single protocols with their merits and challenges discussed extensively elsewhere [7]. In short, the default is a “single protocol” approach (e.g. AddAspirin) with one document to capture all aspects of the trial but sections, particularly the treatment or eligibility section, can become long and unwieldy over time. “Modular protocols” (master plus comparison-specific appendices, e.g. FOCUS4) may circumvent this issue through grouping relevant information but may entail many documents under separate version control which then require careful oversight to ensure the correct documents being used. The choice of protocol structure is best made as the most future-proofed option considering in particular how many new arms are expected (fewer favours a single protocol) and whether sites will take part in all arms (If not, a modular protocol is more user-friendly). In general, the more separate the questions or the more separate the diseases the more likely a modular protocol would be more useful. We recommend explicitly stating in the protocol from the outset that future comparisons will be incorporated into the protocol where appropriate, as this facilitates making such additions via a protocol amendment.

Platform protocols may involve pre-planned interim analyses and the protocol must adequately explain the actions arising from early stopping of recruitment to one or more comparisons and flag any potential for new comparisons. The protocol structure and content should be regularly reviewed for compliance with regulations and good practice: platform protocols will often run for longer than standard 2-arm protocols and are therefore more likely to be exposed to such changes. Eligibility criteria could be common across the whole trial or specific to each treatment comparison. This has implications for the practicalities of recruitment at sites with clarity about which participants can, or do, contribute to which comparisons and should be considered clearly in dissemination of findings and their generalisability.

Many regulators encourage trial teams to discuss all aspects in detail before submitting a platform protocol. Early engagement and ongoing communication with the regulator is essential in these trials [17]. Trialists should aim for review by an ethics committee with previous experience and training in platform protocols [18].

### Database and randomisation system

Thorough consideration is needed for each platform protocol in choosing a database and choosing a structure for the database. Platform protocols require databases with flexible, scalable designs, and capacity to incorporate changes and growth. The randomisation system must cope with stopping and adding arms whilst maintaining the desired randomisation algorithm and varied activation times at individual sites.

Platform trials will employ either a single database or a modular database design with shared elements; the database structure decision can be separate to the decision about protocol structure. A modular approach is likely preferable if the current (or future) arms may differ in terms of the information required, e.g. toxicity or efficacy reporting requirements.

The larger and more complex the platform protocol, the more data management, programmer, and statistical expertise is required to develop and maintain the databases; this may be little recognised by funders. Example challenges in developing case report forms (CRFs) and databases for adaptive trials, with recommendations for possible approaches, are detailed elsewhere [6]. Notable challenges arise from adding or closing comparisons or from updates in the standard-of-care or medical coding dictionaries. Platform protocols may employ generic CRFs for all comparisons, comparison-specific CRFs, or a mix. The right choice will depend on the trial and data in question, which can be elicited by a risk assessment of data collection within and across comparisons; the risk assessment always needs updating when a new comparison is added and is usefully revisited when a comparison is closed out. Collecting data to effectively monitor patient safety should be paramount in all comparisons.

The platform approach also may not necessarily offer savings in central data management despite the shared infrastructure. In PLATO, outcome measures are similar across the three sub-trials but each of them has specific eligibility criteria, interventions, assessment timings, and pharmacovigilance reporting requirements, thus each sub-trial needed dedicated CRFs. The sub-trials differed in target sample size, numbers of treatment arms, and recruitment and analysis periods, and so three separate databases were built and separate randomisation services used. A one-size-fits-all approach is likely inappropriate and anything other than a tailored approach may need to be robustly justified to funders.

Trialists should ensure the choice of randomisation system can incorporate changes to eligibility, sample size, multi-randomisation sub-groups, weightings, and stratification factors.

### Patient and public involvement (PPI)

The importance of PPI in designing and delivering RCTs is increasingly recognised and should be considered vital in platform protocols. For example, the plasmaMATCH (ISRCTN16945804) and PHOENIX (ISRCTN47127434) trial teams sought PPI at an early stage, getting input from multiple patient advocate groups and discussion at targeted forums of patients. Although it is good to have experienced PPI input on oversight committees, it is important to seek views from a broader group. Early involvement of PPI members ensures alignment with the patient pathway and improved acceptability of a complex trial design. PPI members helped the teams produce participant information materials to communicate in accessible language the research aims and activities. By naming the patient advocates as collaborators on the grant applications, teams can ensure continued PPI engagement throughout trial development. The PHOENIX team found PPI members were better able than the trial team to communicate to regulators and ethics committees the acceptability and importance of the novel trial design to patients. Hence, we encourage their attendance at regulatory advisory and ethics committee meetings. PPI engagement must continue as the platform evolves, with PPI representatives supported in understanding the design implications to enable input on the development of new comparisons and closure of existing ones. The support should include clear non-technical communication materials and help from others. PPI input should be appropriately budgeted for in the grant. In larger trials, it may enable better support of the PPI representatives if each PPI member is associated with one of the comparisons to make their workload more manageable.

### Contracts and agreements

Platform protocols assessing investigational agents (CTIMP trials) may require interaction with, and support from, multiple pharmaceutical companies. Contracts may also be needed with further parties (e.g. multiple academic institutions, hospitals, and industry partners) and each needs to encompass the long-term nature of platform trials. This aspect likely takes more time to agree than for any standard two-arm trial. Pushing for a standard template with the same terms and conditions may be valuable, e.g. as in plasmaMATCH. As the importance of genetic profiling increases, in order to define potential pathways within these complex trials, contractual arrangements with laboratories are critical and must be in place before such work is undertaken. Regulatory compliance of all partners should be assured within contractual arrangements, for the protection of patients and to ensure robustness of data. Another option is to have a start-up contract so that the trials unit can get to the stage of ethics and regulatory submission whilst the main contract is being discussed.

### External trial oversight

All late-phase RCTs must choose whether to have an external independent oversight committee (such as Data Monitoring Committees). Long-term protocols will benefit from external oversight committees comprised of researchers with practical experience of running such trials. Charters for such oversight committee may define a committee of standard size and function as in traditional trial designs but the committee should expect greater longevity and to face a greater workload if the trial has many comparisons with separate interim analyses or frequent addition of new arms. A larger committee may help to make the meetings quorate when some members cannot attend. The oversight committees must ensure that recruitment to the protocol is not being hampered by an infeasible or an unpopular comparison. Statistical members must fully understand the original design and the implications of any adaptations. Replacement of committee members is more likely needed in long-term trials or in those with intense meeting and review schedules so handover to new members must be well organised. A summary of historical decisions should ideally be prepared for new members, and the new and departing member should aim to attend at least one meeting together.

### Trial management group

Platform trials are supported by large Trial Management Groups, e.g. FOCUS4’s TMG peaked at 34 members. The responsibilities of such large TMGs can usefully be delegated to specific sub-committees, each responsible for components of the platform. A functional TMG is prepared to adapt its leadership structure, engage the most suitable expertise at each trial stage, and recognise those involved, including through authorship and exposure at conferences. An overall chief investigator (CI) and trial team can be usefully supported by comparison CIs (CCIs) and comparison co-CI (CoCCI) who can play major roles in development, conduct, and reporting of each comparison [7]. Within such a structure, platform trials offer an important opportunity for development of potential CIs of the future. Due the potential for interim decisions, communication channels between trial committees need to be clear and a communication plan should be in place.

### Trial staffing

Like all trials, platform protocols need to be adequately resourced, but prediction of requirements is often more difficult. In traditional trials, tasks usually happen sequentially; in platform protocols, tasks relating to set-up, process improvement, and oversight happen concurrently and continuously as new comparisons are developed, costed, and opened or closed over time [6, 19–22]. Sufficient resources must be available and allocated to oversee safety and data quality in ongoing arms and timely set-up of new comparisons. Site staff will require more support than expected for a traditional trial design. Trials unit staff may need to develop new processes and skill sets to deliver these trials successfully, adding to the resource burden. Succession planning for platform protocol staff is essential, requiring more input from senior staff.

An illustration of this, based on three real trials, is provided in Table 3. PlasmaMATCH approximately aimed to screen 1200 patients to recruit 200 patients into the platform’s therapeutic components, forming five arms. The trial aimed to recruit relatively few participants: three arms required < 16 participants, the others 69 and 78. However, 1.5 full-time equivalent/year (FTE/yr) trial management and 1.5 FTE/yr data management staff were required for delivery. A traditional trial recruiting the same number of participants is likely to require two thirds this staffing. These numbers are not unique: FOCUS4, a platform protocol approximately aimed to screen 1400 to recruit 400, required 2 FTE/yr trial management and 2 FTE/yr data management staff.

**Table 3:** Indicative snapshot in 2019 of the fulltime-equivalent (FTE/yr) staffing levels in two similarly sized platform protocols and a hypothetical traditional trial

**Role**	**Traditional trial (200 randomised)**	**PlasmaMATCH (1200 screened, 200 randomised)**	**FOCUS4 (1400 screened, 400 randomised)**^a^
Senior role	0.35	1	1
Statistician	0.35	1	1
Trial manager	1	1.5	2
Data manager	1	1.5	2
Data scientist/programmer	0.35	1	2
Trial assistant	1	1	1

^a^Approximate numbers given

Larger staffing numbers and greater scientific and operational complexity requires more senior-level project management oversight. PlasmaMATCH allocated 1 FTE/yr senior-level project management staff whilst participants were receiving active treatment, three times that typical for this trials unit for a traditional trial with similar participant numbers. Similarly, FOCUS4 allocated 1 FTE/yr senior-level project management staff to manage the team. These platforms may appear staffing-intensive but are staffing-efficient compared to multiple separate trials and screening programmes assessing the same number of primary hypotheses.

Some trials units may struggle to resource platform protocols appropriately if the recruitment rate is higher than anticipated or new processes or training are required to support the delivery of new trial adaptations. For example, the data management resources allocated in PlasmaMATCH were considered insufficient to manage the rapidly accumulating data whilst adapting the platform to include new arms. Negotiating funding for these posts can prove difficult since commercial funders may wish to focus on funding the comparison relevant to their treatment rather than the totality of the platform. Funders must recognise the scale and complexity of platform designs to ensure sufficient funding is available to support safe, efficient trial and data management activities. Trials units need to develop and maintain ongoing communication with funders, particularly those providing infrastructure costs, as funding changes happen throughout the trial as arms are added and dropped, and other major changes happen.

### Data management

Platform protocols present specific data management challenges requiring bespoke solutions. The numbers of trial arms, participants, and participating centres are likely to be on a much larger scale than for most traditional two-arm trials making the total data volume. Data cleaning and checking should be considered an ongoing process, not an activity performed only prior to analysis, both so as not to overwhelm sites and so that all arms are cleaned with a similar frequency. This avoids any bias when reporting the results from one or many arms. The data cleaning process needs to be set up as a functional system not reliant on a particular person. As the data burden tends to be greater in platform protocols, extra care should be given to minimising the volume of data collected and/or targeting data cleaning at data items that will soon be used. If we consider each comparison as a trial that happens to share its protocol with other trials, the requests to sites are no different within each trial. However, as all requests are under one protocol and platform trials tend to continue for longer, communications can lead a busy site to feel overburdened. Some trials have found it useful to have a dedicated committee across sites and role functions to capture and address site experiences (e.g. “Site Advisory Group”).

### Statistical considerations

One statistical analysis plan may cover the entire platform protocol, e.g. PLATO and CompARE, but this will not be appropriate for every platform protocol; the choice will depend on how similar are the outcome measures, the timing of analyses, and the potential for new analysis methods developed later. Within a platform protocol team, it is important to have a senior statistician involved in analyses during the trial who will attend Independent Data Monitoring Committee (IDMC) meetings and to give consideration to avoiding bias by including a senior statistician, who is unaware of the accumulating data analysis, in decisions around adaptations. Adding arms and changing the control arm present statistical challenges which are discussed elsewhere [23–25], as are design issues [26].

### Safety

In principle, serious adverse event (SAE) processing is the same in platform trials as in other trials. With multiple treatment arms, there are more contemporaneous treatments against which sites (principle investigators) and safety reviewers must assess relatedness and expectedness and trials unit staff likely have more work in ensuring that the correct reference safety information (RSI) is being used. In these trials, with several interventions from different pharmaceutical collaborators for example, there are more parties to choose between to notify of an SAE. It may help to have a dedicated member of staff for managing SAEs, who can develop expertise in this area.

### Training

Due to the potential longevity of platform trials, training materials and documentation for site and trials unit staff will need to be updated regularly. Approaches to training sites need to be kept fresh and inspiring throughout in order to avoid training fatigue. Long-term trials should also benefit from “history training” for new trials unit staff.

Sites might usefully be aware of any forthcoming interim lack-of-benefit analyses and of potential actions that may be needed in response [9]. Streamlined training may be suitable for sites who are demonstrably familiar with the design. Site and CTU staff could usefully understand why recruitment need not be paused around interim analyses.

### Reporting

Although the emphasis for reporting to participants should be on information pertinent to their comparison, many participants will wish to hear about all aspects of the protocol; participants may talk together and it is fairer to transparently offer results from across the protocol’s activities to all participants should they want it [27]. Communicating efficacy results and emerging safety data requires careful tailoring to participants who were in the comparison, participants whose future management in the trial may be impacted by the findings, and participants elsewhere in the platform. Ideally, participants would flag whether they wanted findings from only “their” comparisons, results from other comparisons, or no results. This could potentially be incorporated into the informed consent process.

The minimum necessary information about the overall protocol needed to contextualise recruitment, treatment, follow-up, and analysis considerations should be given. Although CONSORT extensions for multi-arm randomised controlled trials [28] and adaptive trials [29] provide pertinent guidance, a tool for building a bespoke CONSORT-based checklist for each platform protocol publication is needed.

Better guidance also needed on when results of a published comparison should be uploaded to trial registers and better trial register systems are needed to hold these results.

Clear publication plans are required for platform protocols to ensure that all comparisons are reported at the appropriate time whilst minimising clashes of staff time. Publication plans should also ensure that all individuals supporting each comparison and the overall protocol are appropriately credited. At comparison set-up, authorship principles should be codified; authorship and order of authors need not be the same for each comparison. It is important to consider how the release of results from one comparison will impact the ongoing comparisons, for example plasmaMATCH reported 4 cohorts within 3 years of opening to recruitment. Trials need to have plans in place to mitigate the risks around this.

### Adding and closing comparisons

Adding comparisons to a platform is similar to starting a new trial, except that a protocol amendment process can be followed rather than a new application to the Competent Authority or Ethics Committee, and participating sites are already experienced in running most aspects of the platform. Agreement for adding a comparison to a platform is needed from the appropriate oversight committees and sponsor, with assent from sites prior to seeking funding. Working through a checklist for deciding whether to incorporate a new clinical research question into an ongoing protocol is useful (see Table 1*of Schiavone 2019 Trials*) [7]. A new, dedicated comparison chief investigator can share workloads and responsibilities as the clinical lead for a comparison. Once funding is secured, the new registration and randomisation processes should be set-up with new CRFs and updates to the pharmacovigilance system. Added comparisons currently pass through amendment sub-committees at UK ethics committees, but we encourage review by, and attendance at, a full ethics committee meeting [18].

Closing recruitment to the relevant arms of a comparison requires careful planning. The Participant Information Sheet (PIS) need not be amended immediately if recruitment to some arms is stopped at short notice [9].

Trialists should aim to close-out elements of each comparison as soon as practicable rather than leaving all comparisons open, and carefully document this process. This makes implementation easier for sites and potential audit/inspection easier for regulatory inspectors. Removal of references to a treatment arm no longer recruiting will simplify the protocol, but safety information relating to a closed arm (dose reductions, long-term effects, etc.) should remain available to sites as long as any patients remain on treatment. Aspects of closing out a completed comparison may follow normal trial closure and quality assurance guidelines. Most trials units’ close-out processes and regulatory timelines will have been designed for traditional trial designs and may require adaptation. Before submitting an “End-of-Trial Notification” discussion is encouraged with regulators around reporting requirements and ongoing consent issues (with regard to future data and/or sample sharing) and with funders around long-term follow-up of participants. Archiving requirements may exceed the scope of the original funding.

When reporting results of a platform trial across time, it is worth thinking of them as the result of separate trials because sites’ recruitment may vary across a trial’s eligibility spectrum over time. For example, after recruitment completed to the original, chemotherapy-based arms of STAMPEDE, the average age of incoming patients increased—some sites had not been randomising older participants with the same vigour, even without age-based eligibility criteria [30]. Other elements of follow-up and assessment can also change over time with changes in needs of active comparisons.

### Maintaining relevant control arm treatment

Randomised trials need to always compare against an up-to-date standard-of-care. In long-running platform protocols, new data may emerge that leads to changes in the standard-of-care for ongoing comparisons. Each research treatment should be assessed against the relevant concurrent standard-of-care at the point of recruitment. In a platform protocol, the data leading to such a change may come from another comparison within the platform and/or from other trials. STAMPEDE has updated its standard-of-care five times, once for an eligibility subset based on data from another trial, once for a subset of patients based on results from a STAMPEDE comparison, and thrice based on a combination of STAMPEDE and other trial results.

### Onward data sharing and re-use

Data and sample sharing are important aspects of modern clinical trials. It is important to recognise that data sharing applications are likely to come in for comparisons with primary results reported whilst other comparisons are still ongoing and so may feel more burdensome. There is an onus on applicants to be clear on exactly which data are required from which comparison. Sharing data from completed comparisons must not compromise ongoing or future work in the platform—applicants and researchers should consider any contractual arrangements with funders and any obligations to report a comparison before data is shared. Funders, of which a platform protocol may have more than a traditional protocol, may have contrasting requirements and platform protocols are more likely to experience this.

## Discussion

The statistical issues underpinning these late-phase trials testing multiple primary hypotheses have been increasingly well-characterised [1], but practical experience remains limited to the relatively few groups with established examples. Trials of this type also remain novel for other stakeholders and these practicalities should also aid funding panels, ethics committees, and regulators. Our aim was to use our collective conduct experiences to enable others conducting such trials to anticipate and address issues specific to such trial designs.

Platform protocol designs allow multiple research questions to be asked within one master protocol and offer time, resource, and/or cost savings. A platform trial means one initial grant submission, one ethics committee, and one set of trial oversight committees. The approval process for each participating country needs to be followed. Platform protocols produce more results in a shorter time frame compared to separate two-arm trials.

Platform protocols have important efficiencies [15, 16] and notable practical challenges. For example, although researchers only need one protocol/set of approvals, amendments can take much longer to implement because of all of the groups that need to agree (multiple funders and oversight committees, etc.). The large staffing requirements for platform protocols can pose a risk to a trials unit and staff redeployment could be challenging should the overall protocol stop prematurely. Some processes do not accelerate as the trial proceed,s e.g. external factors affecting the speed of opening a new comparison may not dissipate just because the trial has previously added arms.

There is no single answer to the conduct questions posed by each platform protocol. For example, the choice whether the protocol should be organised as one document or nested, connected documents, or the choice whether comparison-specific CRFs are preferred or a core CRF set supplemented by extra CRFs when required will depend heavily on the particular platform protocol’s needs and the team’s implementation preferences.

Platform protocols provide an excellent opportunity to support the progression of junior academic, scientific, and clinical staff through prolonged involvement into senior roles. Trials units with experience in platform protocols have supported the development of less-experienced trials units through design and/or membership of oversight committees.

We have deliberately limited our focus to UK-based experiences and late-phase trials. Many principles will translate internationally and to earlier phase platform protocols, including non-randomised comparisons, and to biomarker-based trials. Our examples are in oncology because this is the first area to embrace a wide range of platform protocols. However, the practical issues discussed are not limited to any one diseases area. There appears to be increasing interest in implementing platform protocols. The COVID-19 pandemic has seen the rapid development and deployment of late-phase platform protocols that assess multiple strategies to manage this new condition. These include the RECOVERY [31], and PRINCIPLE trials [32] in the UK and the WHO SOLIDARITY and TICO trial internationally [33, 34]. With their very fast recruitment rates and rapid collection of short-term efficacy outcome measures and with their support by, and involvement from, national prioritisation committees, including around the incorporation and assessment of new agents, the challenges faced by those trials may differ from the examples we have drawn on. The EU-PEARL group [35] is seeking to develop guidance on planning, implementation and analysis of earlier phase platform protocols, and the Clinical Trials Transformation Initiative (CTTI) have developed some practical resources for platform protocol design and conduct [36]. We encourage the sharing of experiences from those trials when clinical and operational pressures permit.

## Conclusions

Platform protocols testing multiple primary hypotheses enable many questions to be answered efficiently to the benefit of patients. Practical lessons from running platform trials will support trial teams in delivering these trials more effectively and efficiently.

## Supplementary Information

**Additional file 1.** Trials whose staff experiences added to this paper.

## Data Availability

No new data.

## Abbreviations

CI: Chief investigator
CCI: Comparison chief investigator
CoCCI: Comparison co-chief investigator
CRF: Case report form
CTIMP: Clinical Trial of an Investigational Medicinal Product
CTTI: Clinical Trials Transformation Initiative
FTE/yr: Full-time equivalent per year
HRA: Health Research Authority
IDMC: Independent Data Monitoring Committee
MAMS: Multi-arm multi-stage
NCRI: National Cancer Research Institute
PIS: Patients/Participant Information Sheet
PPI: Patient and public involvement
R&D: Research and development
RCT: Randomised controlled trial
RSI: Reference safety information
SAE: Serious adverse event
TMG: Trial Management Group
UKCRC: United Kingdom Clinical Research Collaboration
