# Supplementary material for: Practical guidance for running late-phase platform protocols for clinical trials: lessons from experienced UK clinical trials units
Source: Trials. 2022 Sep 6;23:757. doi: 10.1186/s13063-022-06680-4 (PMC9449272; doi:10.1186/s13063-022-06680-4)

# Supplementary file 1

# Appendix 1 –trials whose staff experiences added to this paper

| **Trial Name** | **Page number** |
| --- | --- |
| AddAspirin | 2 |
| AML LI-1 | 3 |
| ATLANTIS | 4 |
| Brain Matrix | 5 |
| CompARE | 6 |
| FLAIR | 7 |
| FOCUS4 | 8 |
| National Lung Matrix Trial | 9-10 |
| OCTOPUS | 11-12 |
| PHOENIX | 13-14 |
| plasmaMATCH | 15 |
| PLATO | 16-17 |
| PrecisionPanc | 18 |
| STAMPEDE | 19 |
| TASTER | 20 |

| **Acronym** | **AddAspirin** |
| --- | --- |
| **Trial name** | A phase III, double-blind, placebo-controlled, randomised trial assessing the effects of aspirin on disease recurrence and survival after primary therapy in common non-metastatic solid tumours |
| **Registration links** | ISRCTN74358648 |
| **Disease setting** | Breast, colorectal, upper GI and prostate cancer. Participants have undergone primary treatment with curative intent |
| **Current status** | Recruiting International (UK, Ireland and India) |
| **Reason for inclusion as example** | Phase III basket trial with four parallel cohorts |
| **Coordinating trials unit**  **Website** | MRC Clinical Trials Unit at UCL, London  <http://www.addaspirintrial.org/> |
| **Flow chart** |  |
|  |  |
| 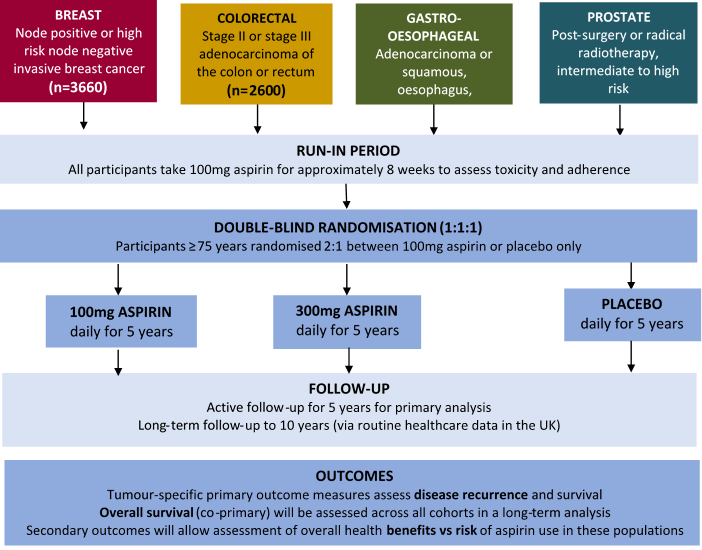 | |

| **Acronym** | **AML LI-1** – a platform trial |
| --- | --- |
| **Trial name** | (as acronym) |
| **Registration links** | ISRCTN40571019 |
| **Disease setting** | Acute Myeloid Lymphoma (AML) patients over the age of 60 and not fit for intensive chemotherapy |
| **Current status** | Closed to recruitment |
| **Reason for inclusion as example** | Adaptive multi-arm pick-a-winner phase II/III trial |
| **Coordinating trials unit**  **Website** | Centre for Trials Research, Cardiff  <https://www.cardiff.ac.uk/centre-for-trials-research/research/studies-and-trials/view/aml-li-1> |
| **Flow chart** |  |
|  |  |
| 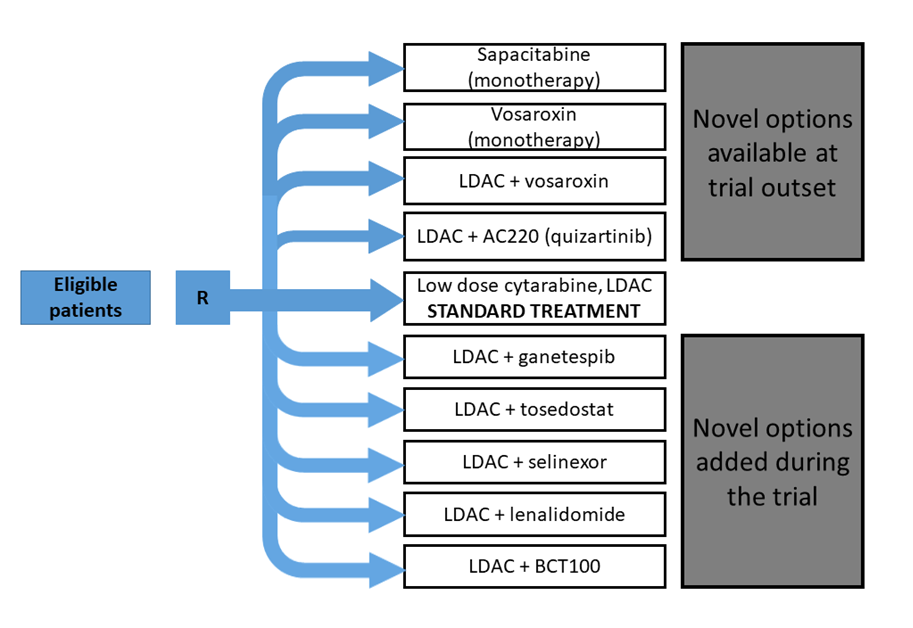 | |

| **Acronym** | **ATLANTIS** |
| --- | --- |
| **Trial name** | An adaptive multi-arm phase II trial of maintenance targeted therapy after chemotherapy in advanced/metastatic urothelial cancer |
| **Registration links** | ISRCTN25859465 |
| **Disease setting** | maintenance targeted therapy after chemotherapy in advanced/ metastatic urothelial cancer |
| **Current status** | Recruiting |
| **Reason for inclusion as example** | Umbrella screening trial with an adaptive multi-arm phase II |
| **Coordinating trials unit** | CRUK Clinical Trials Unit Glasgow |
| **Website** | - |
| **Flow chart** |  |
|  |  |
| 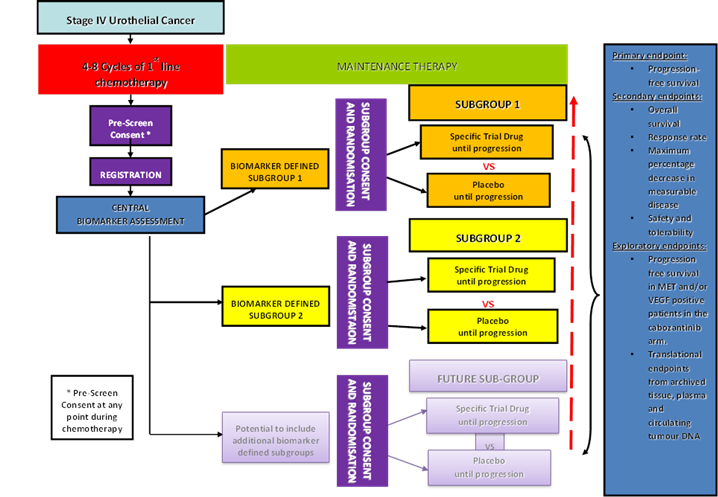 | |

| **Acronym** | **Tessa Jowel BRAIN MATRIX** |
| --- | --- |
| **Trial name** | A BRitish feasibility study of molecular stratification and targeted therapy to optimize the clinical mAnagement of patIeNts with glioMA by enhancing clinical ouTcomes, Reducing avoIdable toXicity, improving management of postoperative residual & recurrent disease and improving survivorship |
| **Registration links** | ISRCTN14218060 |
| **Disease setting** | Glioma |
| **Current status** | Recruiting International (UK, Ireland and India) |
| **Reason for inclusion as example** | Platform protocol |
| **Coordinating trials unit**  **Website** | CRUK Clinical Trials Unit, Birmingham  <https://www.birmingham.ac.uk/research/crctu/trials/brain-matrix/index.aspx> |
| **Flow chart** |  |
|  |  |
|  | |


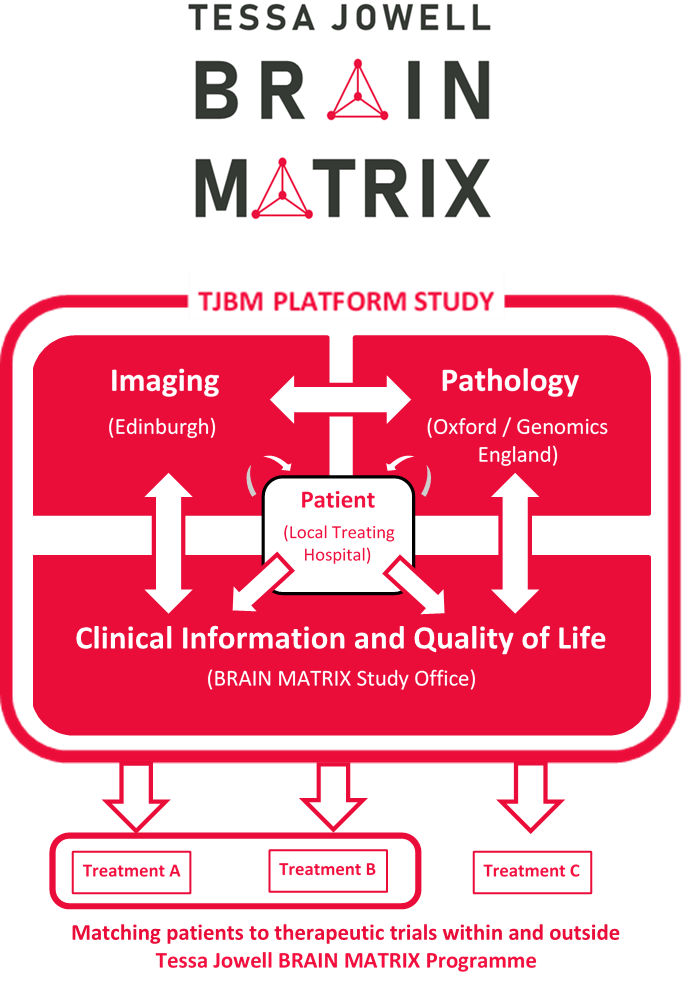


##

| **Acronym** | **CompARE** |
| --- | --- |
| **Trial name** | A phase III randomised controlled trial Comparing Alternative Regimens for escalating treatment of intermediate and high-risk oropharyngeal cancer |
| **Registration links** | ISRCTN4148539 |
| **Disease setting** | Oropharyngeal cancer |
| **Current status** | Recruiting International (UK, Ireland and India) |
| **Reason for inclusion as example** | Phase III MAMS trial |
| **Coordinating trials unit**  **Website** | CRUK Clinical Trials Unit, Birmingham  <https://www.birmingham.ac.uk/research/crctu/trials/compare/index.aspx> |
| **Flow chart** |  |


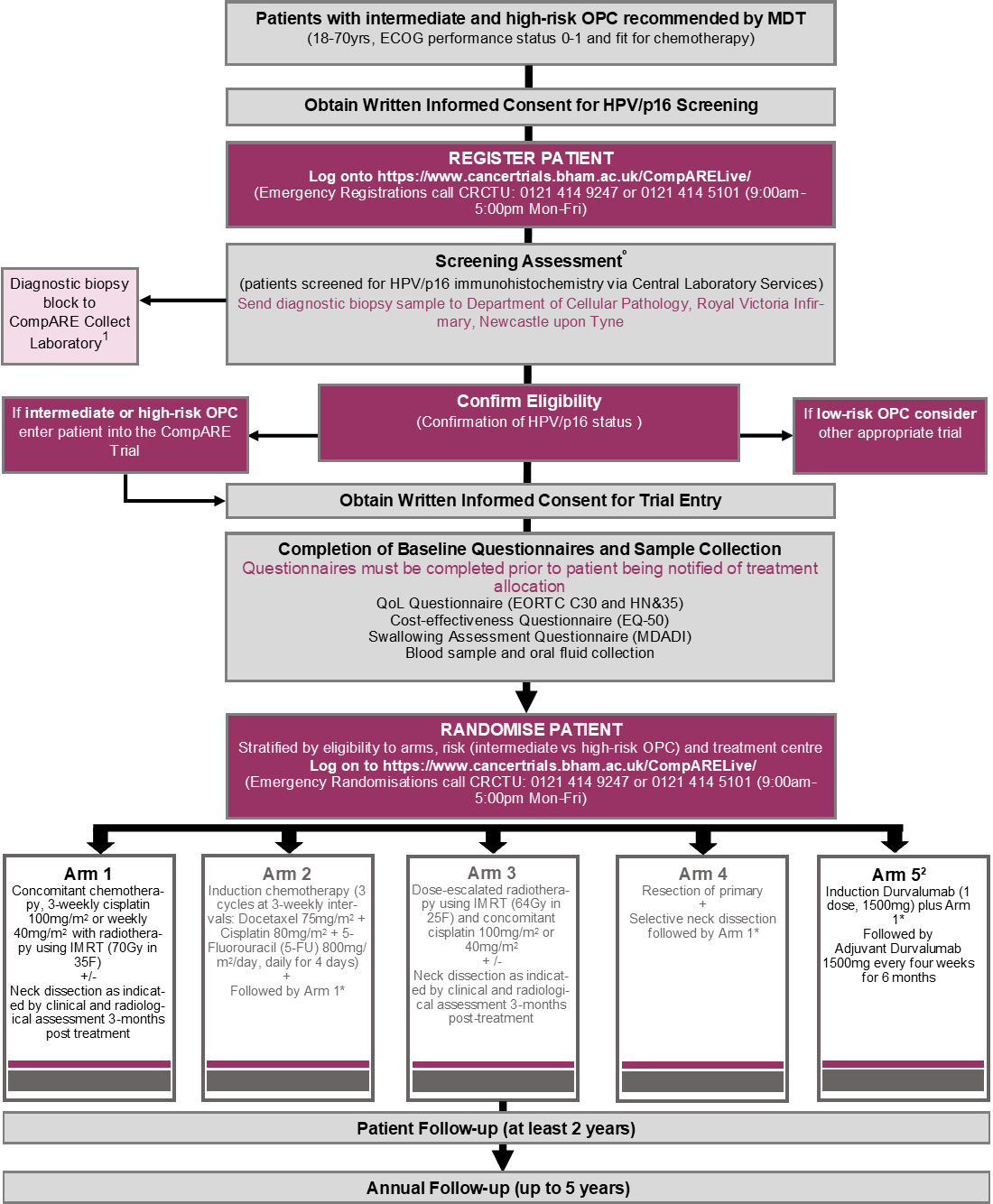


| **Acronym** | **FLAIR** |
| --- | --- |
| **Trial name** | Front-Line therapy in CLL: Assessment of Ibrutinib-containing Regimes:  a randomised controlled trial |
| **Registration links** | ISRCTN01844152 |
| **Disease setting** | CLL |
| **Current status** | Recruiting high risk |
| **Reason for inclusion as example** | Phase III platform |
| **Coordinating trials unit**  **Website** | Leeds Institute of Clinical Trials Research  - |
| **Flow chart** |  |
| 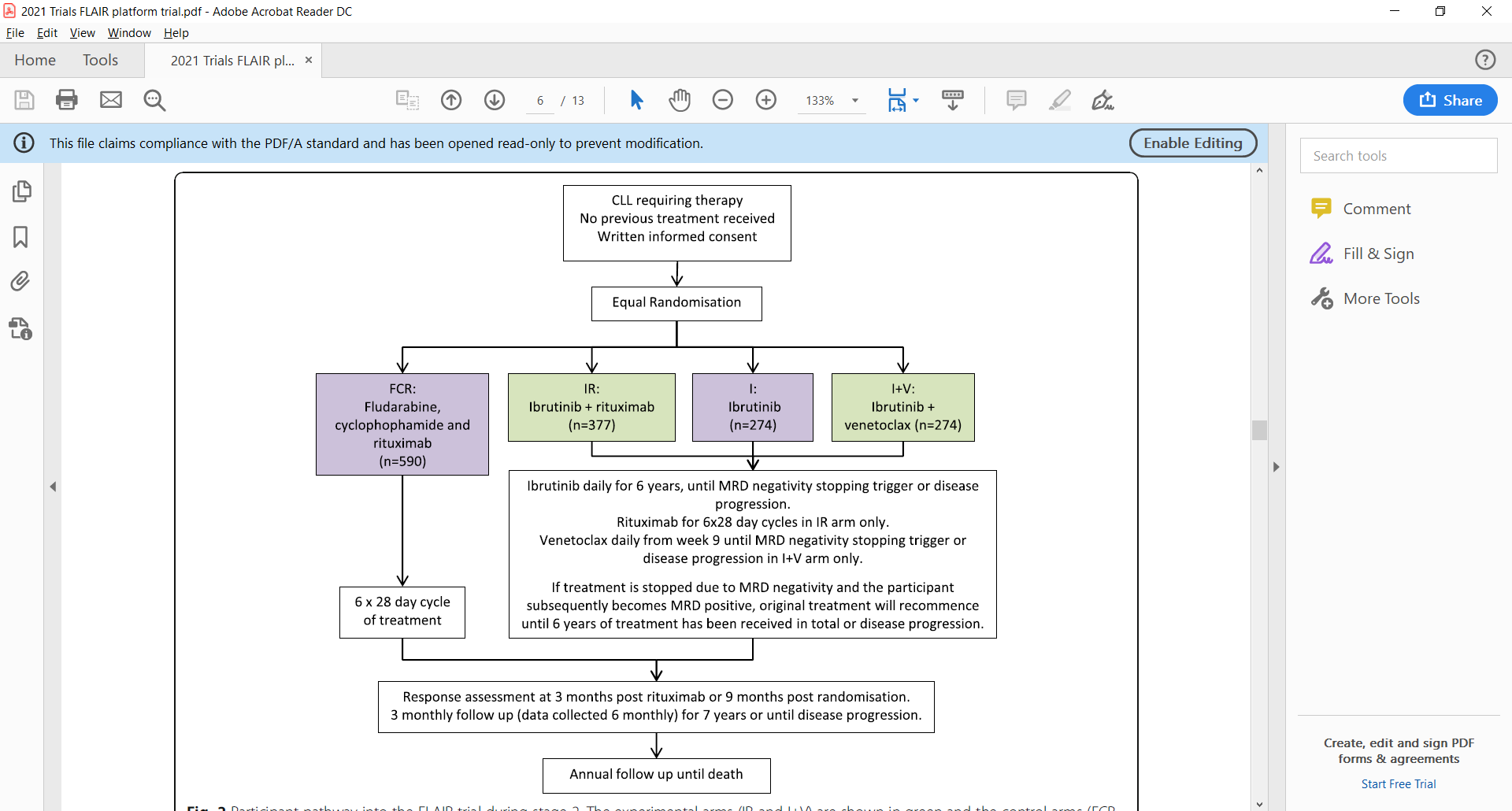 | |
|  | |

| **Acronym** | **FOCUS4** |
| --- | --- |
| **Trial name** | FOCUS4 is a molecularly stratified, multi-site randomised trial programme for patients with colorectal cancer |
| **Registration links** | ISRCTN90061546 |
| **Disease setting** | Colorectal cancer |
| **Current status** | In follow-up |
| **Reason for inclusion as example** | A master protocol umbrella design |
| **Coordinating trials unit**  **Website** | MRC Clinical Trials Unit at UCL, London  <http://www.focus4trial.org/> |
| **Flow chart** |  |

| **Acronym** | **National Lung Matrix Trial** |
| --- | --- |
| **Trial name** | Multi-drug, genetic marker-directed, non-comparative, multi-centre, multi-arm phase II trial in non-small cell lung cancer |
| **Registration links** | ISRCTN38344105 |
| **Disease setting** | Non-small cell lung cancer |
| **Current status** | Recruiting |
| **Reason for inclusion as example** | Umbrella study |
| **Coordinating trials unit**  **Website** | CRUK Clinical Trials Unit, Birmingham  <https://www.birmingham.ac.uk/research/crctu/trials/lung-matrix/index.aspx> |
| **Flow chart on next page** |  |
|  |  |
|  | |


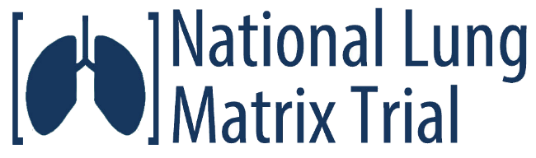

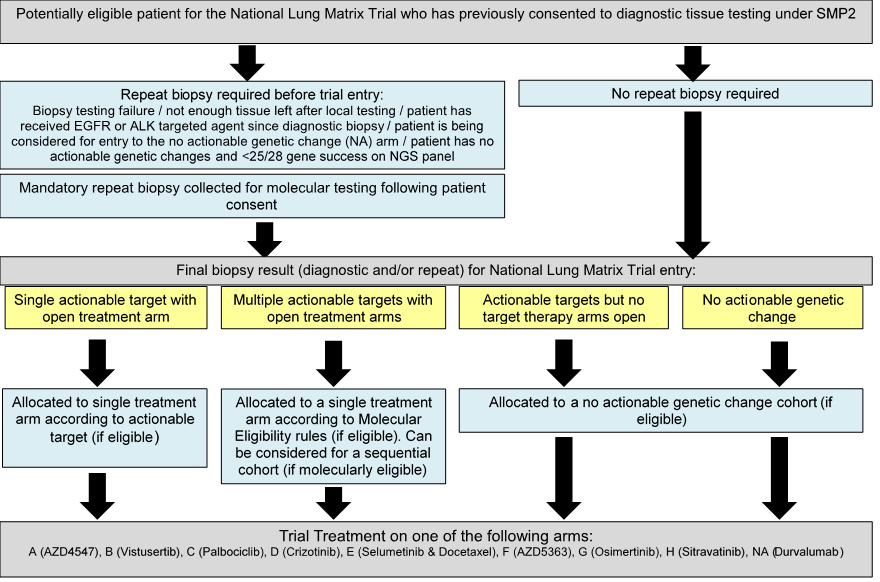

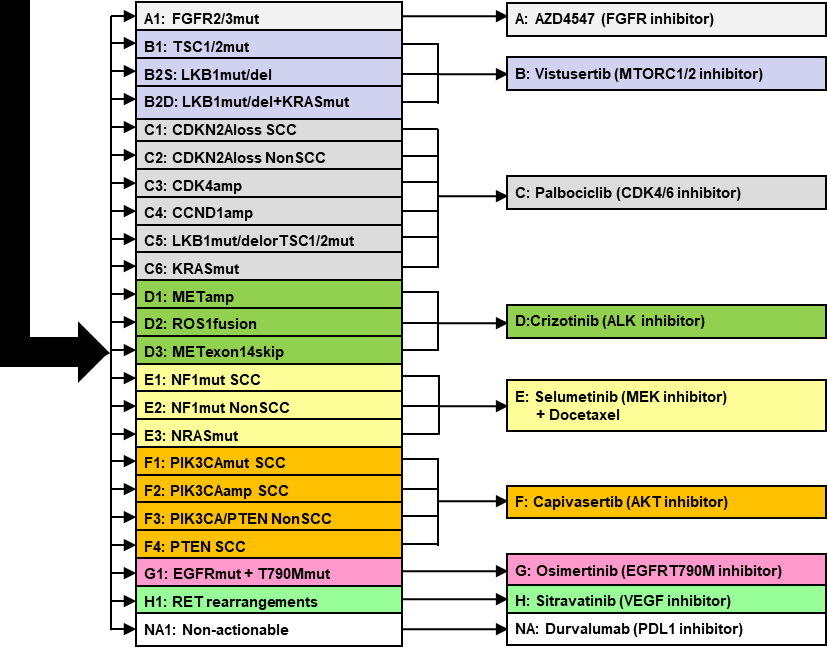


Middleton, G., Fletcher, P., Popat, S. *et al.* The National Lung Matrix Trial of personalized therapy in lung cancer. *Nature* **583,**807–812 (2020). [https://doi.org/10.1038/s41586-020-2481-8](https://eur01.safelinks.protection.outlook.com/?url=https%3A%2F%2Fdoi.org%2F10.1038%2Fs41586-020-2481-8&data=02%7C01%7C%7C17846f09f8094f7f335c08d84397c249%7C1faf88fea9984c5b93c9210a11d9a5c2%7C0%7C0%7C637333666385717400&sdata=SeJybvhUGV2y2AlUDndE1IVTS%2B3XSWxB2h3DwH4XOSU%3D&reserved=0)

| **Acronym** | **OCTOPUS** |
| --- | --- |
| **Trial name** | A Randomised, Phase II Umbrella Trial of Weekly Paclitaxel +/- Novel Agents in Platinum-Resistant Ovarian Cancer |
| **Registration links** | ISRCTN16426935 |
| **Disease setting** | Breast, colorectal, upper GI and prostate cancer. Participants have undergone primary treatment with curative intent |
| **Current status** | In follow-up |
| **Reason for inclusion as example** | An umbrella trial |
| **Coordinating trials unit**  **Website** | CRUK Clinical Trials Unit Glasgow  - |
| **Flow chart on next page** |  |
|  |  |
|  | |

**
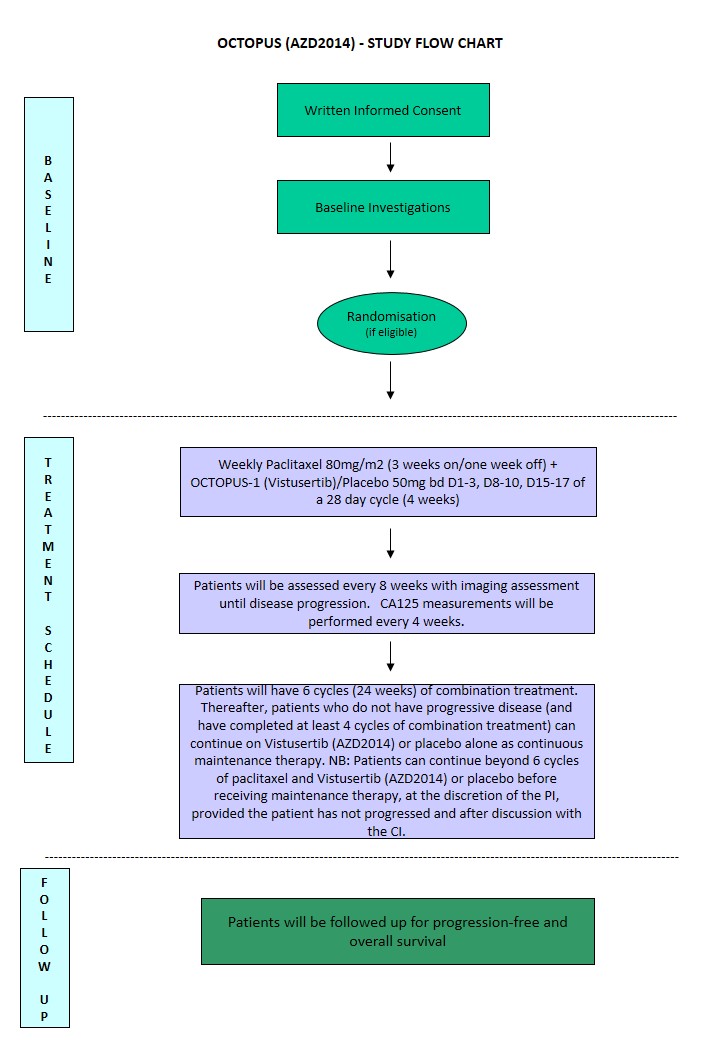
**

| **Acronym** | **PHOENIX** |
| --- | --- |
| **Trial name** | A pre-surgical window of opportunity and post-surgical adjuvant biomarker study of DNA damage response inhibition and/or anti-PD-L1 immunotherapy in patients with neoadjuvant chemotherapy resistant residual triple negative breast cancer. |
| **Registration links** | ISRCTN47127434 |
| **Disease setting** | Breast cancer |
| **Current status** | Recruiting |
| **Reason for inclusion as example** | A platform trial |
| **Coordinating trials unit**  **Website** | ICR-CTSU, Sutton  <https://www.icr.ac.uk/our-research/centres-and-collaborations/centres-at-the-icr/clinical-trials-and-statistics-unit/clinical-trials/phoenix> |
| **Flow chart on next page** |  |
|  |  |
|  | |


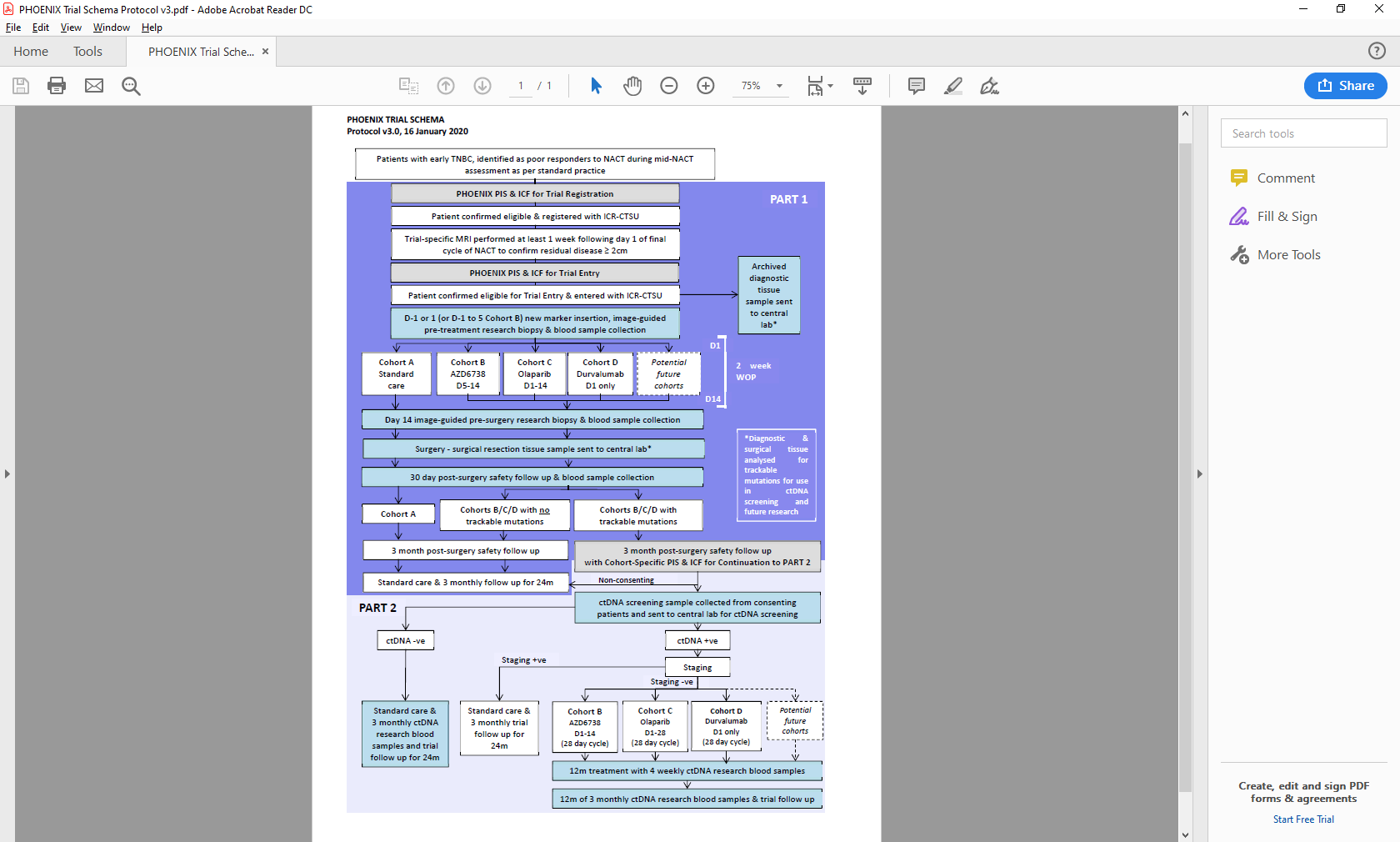


| **Acronym** | **plasmaMATCH** |
| --- | --- |
| **Trial name** | A multiple parallel cohort, open-label, multicentre phase IIa clinical trial aiming to provide proof of principle efficacy for designated targeted therapies in patients with advanced breast cancer where the targetable mutation is identified through ctDNA screening |
| **Registration links** | ISRCTN16945804 |
| **Disease setting** | Breast cancer |
| **Current status** | Closed |
| **Reason for inclusion as example** | An umbrella trial |
| **Coordinating trials unit**  **Website** | ICR-CTSU, Sutton  <https://www.icr.ac.uk/our-research/centres-and-collaborations/centres-at-the-icr/clinical-trials-and-statistics-unit/clinical-trials/plasmamatch> |
| **Flow chart** |  |


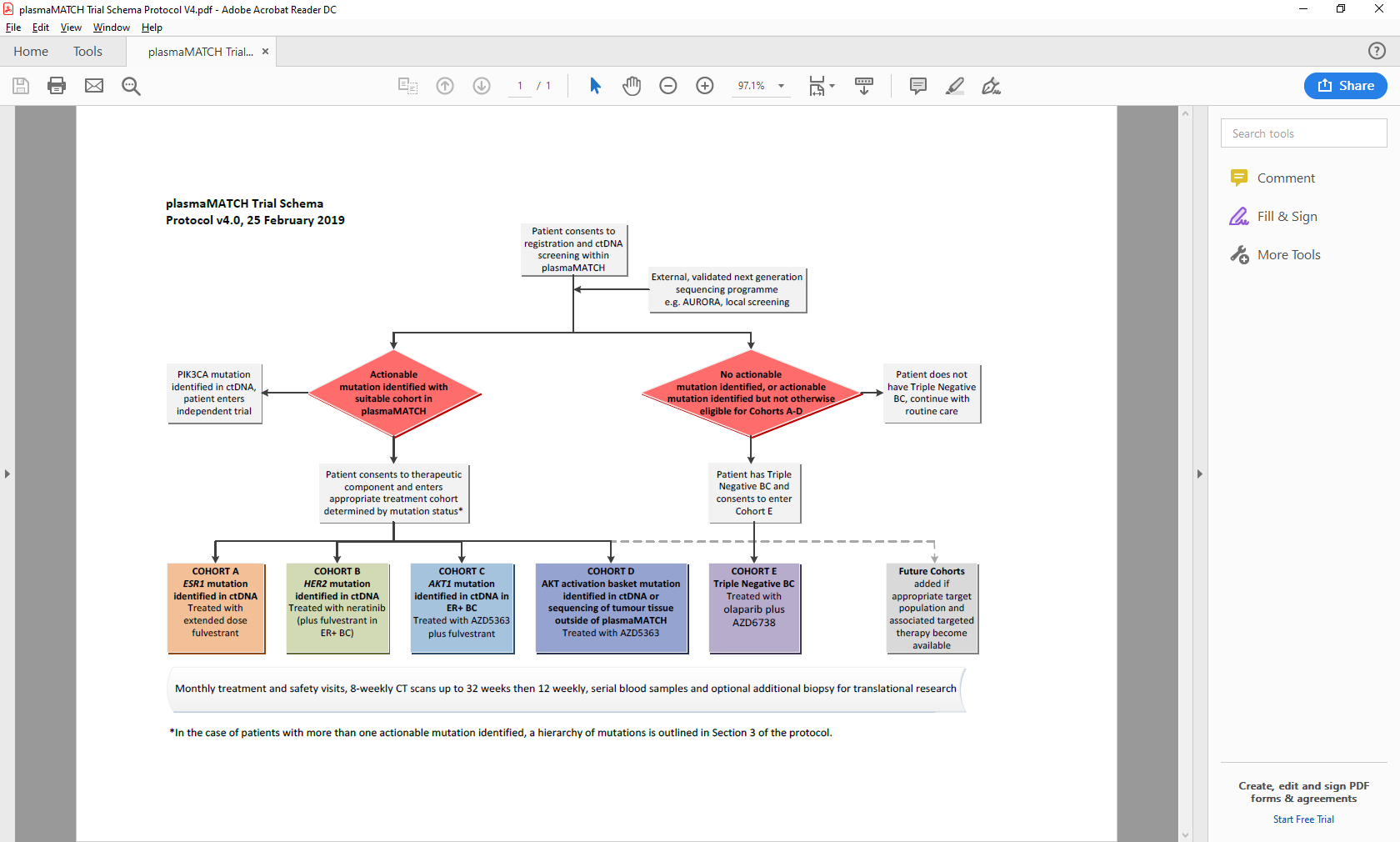


| **Acronym** | **PLATO** |
| --- | --- |
| **Trial name** | PersonaLising Anal cancer radiotherapy dOse |
| **Registration links** | ISRCTN88455282 |
| **Disease setting** | Anal cancer |
| **Current status** | Recruiting |
| **Reason for inclusion as example** | An umbrella trial with platform elements |
| **Coordinating trials unit**  **Website** | Leeds Institute of Clinical Trials Research  - |
| **Flow chart on the next page** |  |
|  |  |
|  | |


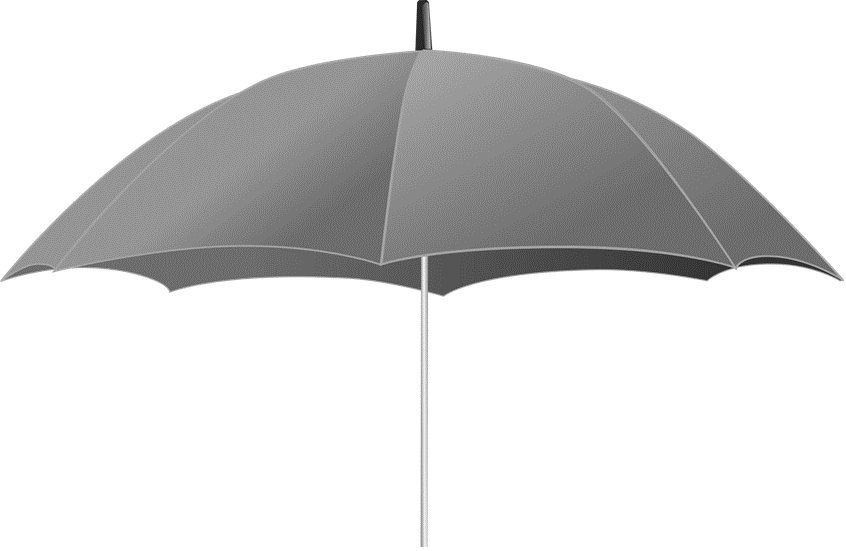


**PLATO**

**P**ersona**L**ising **A**nal cancer radio**T**herapy d**O**se

ACT3

Low-risk disease

ACT4

Intermediate-risk disease

ACT5

High-risk disease

Standard treatment strategy

Dose de-escalation

Dose escalation

- Study of multiple targeting therapies in parallel in a single disease
- Disease is classified into different subgroups, depending on the presence of a particular biomarker/mutation, disease stage, and risk groups, for example
- One overarching protocol was designed to answer multiple research questions


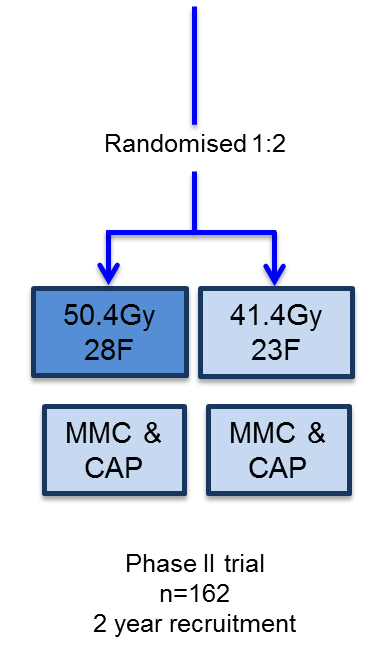

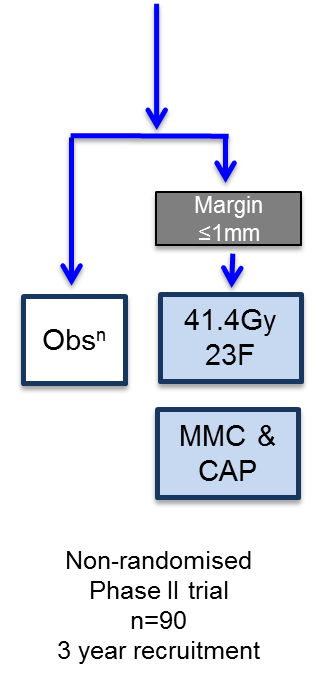


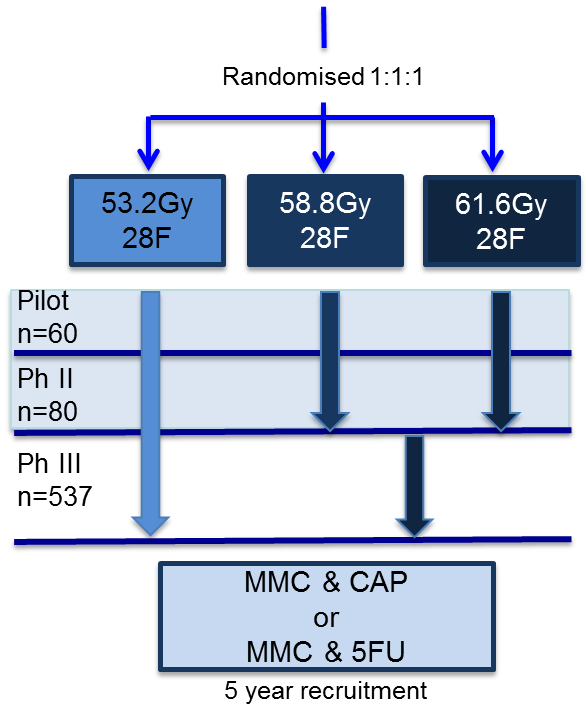


n=500

ACT5

ACT4

ACT3

Intermediate-risk disease

High-risk disease

Low-risk disease

| **Acronym** | **Precision-Panc** |
| --- | --- |
| **Trial name** | The Precision-Panc Master Protocol is a “portal” protocol for patients with known or suspected pancreatic cancer to be accrued through multiple centres in the UK, with the option of being subsequently recruited into PRIMUS (Pancreatic canceR Individualised Multi-arm Umbrella Studies) examining different treatment regimens and/or biomarker development. |
| **Registration links** | ISRCTN14879538 |
| **Disease setting** | Pancreatic cancer |
| **Current status** | Recruiting |
| **Reason for inclusion as example** | Master protocol |
| **Coordinating trials unit**  **Website** | CRUK Clinical Trials Unit Glasgow  <https://www.precisionpanc.org/> |
| **Flow chart** |  |


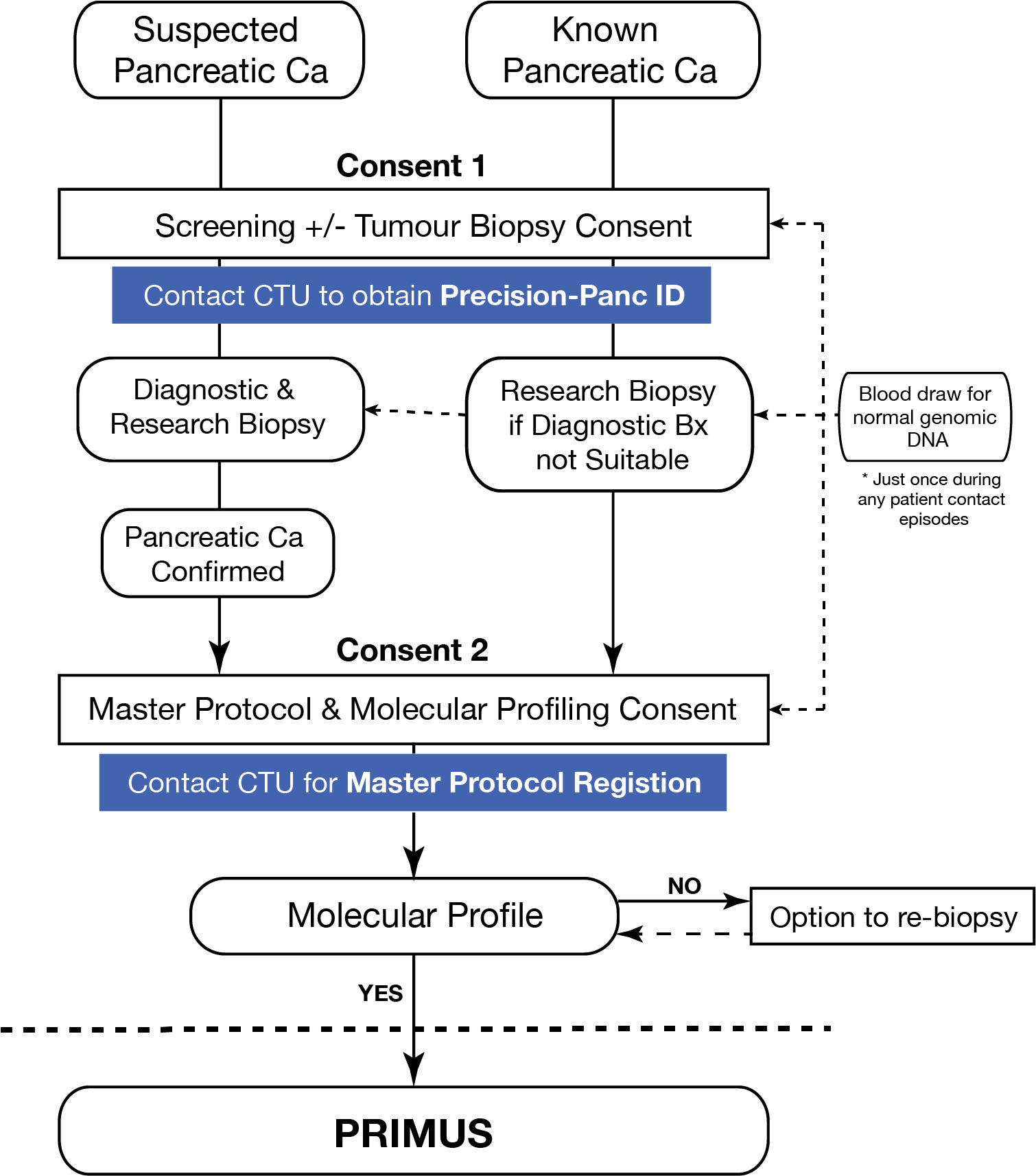


| **Acronym** | **STAMPEDE** |
| --- | --- |
| **Trial name** | **S**ystemic **T**herapy in **A**dvancing or **M**etastatic **P**rostate **C**ancer: **E**valuation of **D**rug **E**fficacy |
| **Registration links** | ISRCTN78818544 |
| **Disease setting** | Prostate cancer |
| **Current status** | Recruiting |
| **Reason for inclusion as example** | Phase III MAMS trial |
| **Coordinating trials unit**  **Website** | MRC Clinical Trials Unit at UCL, London  <http://www.stampedetrial.org/> |
| **Flow chart** |  |

| 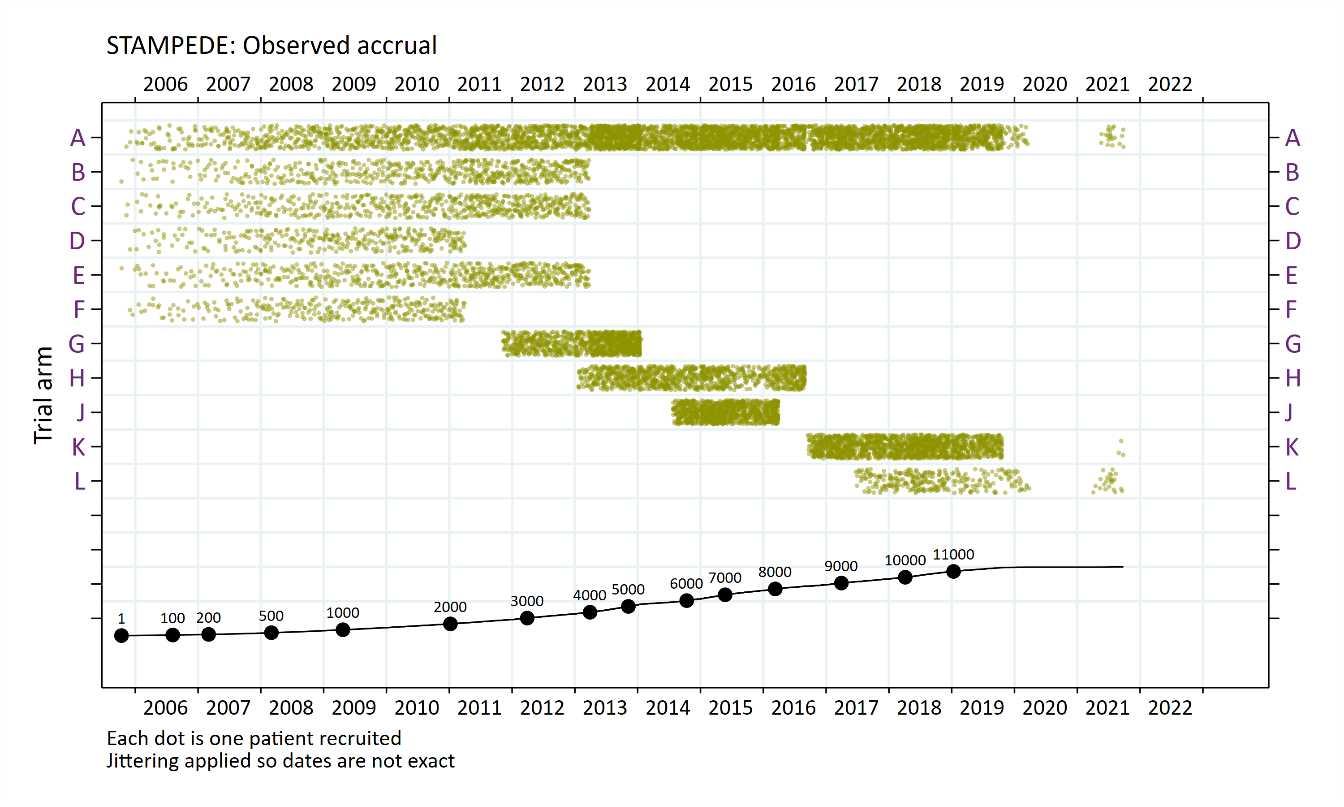 | |
| --- | --- |
| **Key to trial arms**  A = Standard-of-care (SOC)  B = SOC+zoledronic acid  C = SOC+docetaxel  D = SOC+celecoxib  E = SOC+zoledronic acid+docetaxel  F = SOC+zoledronic acid+celecoxib | G =SOC+abiraterone  H =SOC+radiotherapy  J = SOC+enzalutamide+abiraterone  K = SOC+metformin  L = SOC+transdermal oestradiol |

| **Acronym** | **Taster** |
| --- | --- |
| **Trial name** | An umbrella adaptive randomised multi-arm screening phase II trial for patients with 2nd / 3rd generation TKI resistant chronic myeloid leukaemia in chronic or accelerated phase |
| **Registration links** | ISRCTN68270067 |
| **Disease setting** | CML |
| **Current status** | On hold |
| **Reason for inclusion as example** | An umbrella screening trial |
| **Coordinating trials unit**  **Website** | CRUK Clinical Trials Unit Glasgow  - |
| **Flow chart** |  |

##
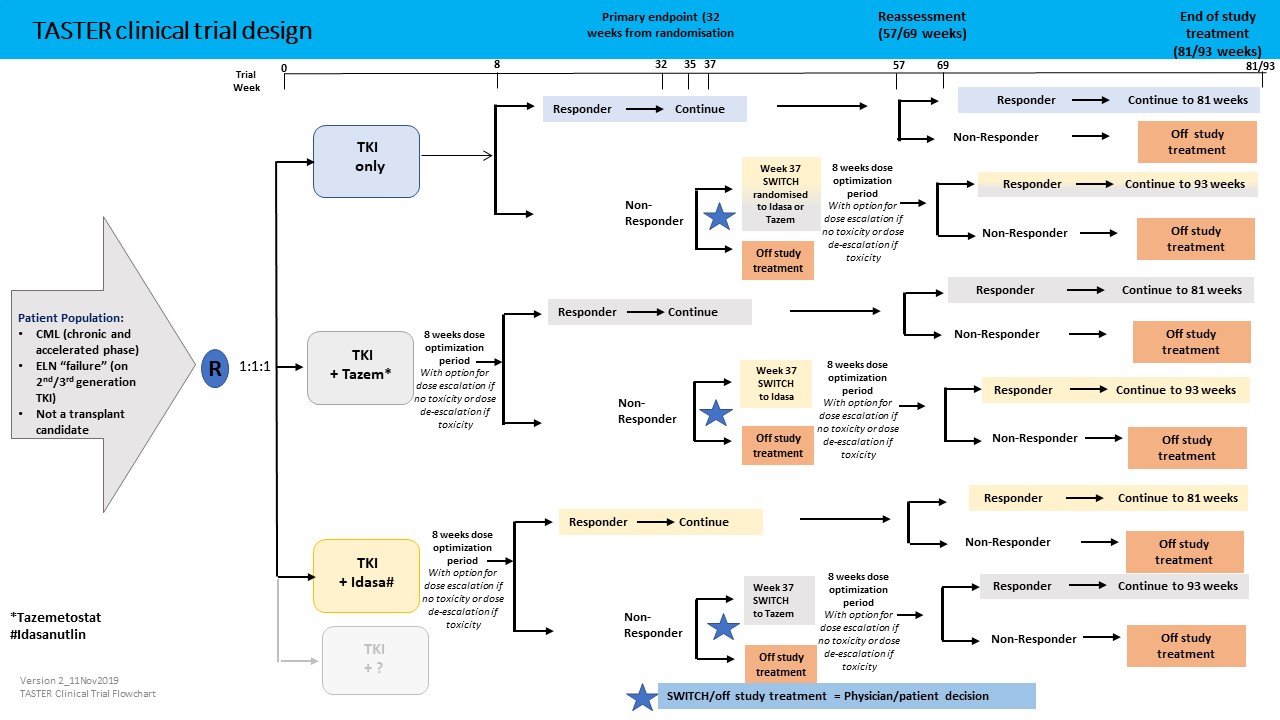

Supplement: Supplementary file 1 — Additional file 1. Trials whose staff experiences added to this paper. [file 13063_2022_6680_MOESM1_ESM.docx]
